# Systematic Evaluation of Voriconazole Pharmacokinetic Models without Pharmacogenetic Information for Bayesian Forecasting in Critically Ill Patients

**DOI:** 10.3390/pharmaceutics14091920

**Published:** 2022-09-10

**Authors:** Simon Kallee, Christina Scharf, Lea Marie Schatz, Michael Paal, Michael Vogeser, Michael Irlbeck, Johannes Zander, Michael Zoller, Uwe Liebchen

**Affiliations:** 1 Department of Anesthesiology, University Hospital, LMU Munich, 81377 Munich, Germany; 2 Department of Pharmaceutical and Medical Chemistry, Clinical Pharmacy, University of Muenster, 48149 Muenster, Germany; 3 Institute of Laboratory Medicine, University Hospital, LMU Munich, 81377 Munich, Germany; 4 Laboratory Dr. Brunner, Luisenstr. 7e, 78464 Konstanz, Germany

**Keywords:** precision medicine, antifungal, model informed precision dosing, pharmacokinetics, therapeutic drug monitoring, voriconazole, population pharmacokinetics, pharmacometrics, critically ill, invasive aspergillosis

## Abstract

Voriconazole (VRC) is used as first line antifungal agent against invasive aspergillosis. Model-based approaches might optimize VRC therapy. This study aimed to investigate the predictive performance of pharmacokinetic models of VRC without pharmacogenetic information for their suitability for model-informed precision dosing. Seven PopPK models were selected from a systematic literature review. A total of 66 measured VRC plasma concentrations from 33 critically ill patients was employed for analysis. The second measurement per patient was used to calculate relative Bias (rBias), mean error (ME), relative root mean squared error (rRMSE) and mean absolute error (MAE) (i) only based on patient characteristics and dosing history (a priori) and (ii) integrating the first measured concentration to predict the second concentration (Bayesian forecasting). The a priori rBias/ME and rRMSE/MAE varied substantially between the models, ranging from −15.4 to 124.6%/−0.70 to 8.01 mg/L and from 89.3 to 139.1%/1.45 to 8.11 mg/L, respectively. The integration of the first TDM sample improved the predictive performance of all models, with the model by Chen (85.0%) showing the best predictive performance (rRMSE: 85.0%; rBias: 4.0%). Our study revealed a certain degree of imprecision for all investigated models, so their sole use is not recommendable. Models with a higher performance would be necessary for clinical use.

## 1. Introduction

Up to 20% of microbiologically documented infections in critically ill patients are caused by fungi [1]. The triazole voriconazole (VRC) is an antifungal agent used as first line treatment against invasive aspergillosis as recommended by the Infectious Diseases Society of America (IDSA) guideline [2]. In addition, it is used for the treatment of candidemia, esophageal candidiasis and infections with scedosporium apiospermum and fusarium species [1,2]. According to Food and Drug Administration (FDA) recommendation, VRC should be administered with a loading dose of 6 mg/kg intravenous infusion every 12 h (q12) on the first day and then maintained with intravenous doses between 3 and 4 mg/kg q12 or 200 mg oral q12. However, physiological alterations and several drug–drug interactions lead to a high interindividual pharmacokinetic variability, especially in critically ill patients [3,4]. VRC is metabolized in the liver and the cytochrome P450 isoenzymes CYP3A4, CYP2C19 and CYP2C9 are involved in the metabolism [5]. Therefore, therapeutic drug monitoring (TDM) is recommended by the IDSA and the British Society of Medical Mycology for optimal treatment and limitation of toxicity [2,6]. There is a significantly lower response rate (clinical and radiological parameters) when plasma trough concentrations are too low (<1 mg/L), while supratherapeutic plasma trough concentrations (>5.5 mg/L) increase the rate of neurotoxicity, visual disturbances, and hepatoxicity [6,7,8]. The benefits of TDM have been confirmed in a randomized trial by Park et al. [9].

Although TDM offers advantages, it is costly, not always available and cannot give dosing advice for the first doses, i.e., before the first sample is taken. The employment of population pharmacokinetic (PopPK) models enables the visualization and optimization of the concentration time profile using the dosing history of the drug and patient characteristics. This allows the calculation of the optimal dosage right from the start. However, the precision of these models has recently been described as insufficient [10]. The inclusion of a single plasma sample in the PopPK models by using Bayesian forecasting potentially leads to more accurate predictions and the frequency of TDM measurements can possibly be reduced compared to traditional TDM (i.e., without model-based dosing approaches) [11]. In patients receiving vancomycin, a benefit of Bayesian forecasting has already been described resulting in less toxic events and the optimization of plasma concentrations [12,13]. Several pharmacokinetic models have been developed for VRC, but none of these models was externally evaluated in critically ill patients, a special population with possibly altered pharmacokinetics [14]. Cheng et al. recently recommended to focus on the generalizability and transferability of PopPK models by externally evaluating PopPK models [15]. Since pharmacogenetic patient characteristics are not universally and routinely available, the aim of the present study was to assess the predictive performance of previously published PopPK models not specifying pharmacogenetic information in critically ill patients. In particular, it should be examined whether adequate predictions can be achieved using Bayesian forecasting and if existing models (without additional model manipulation) might be suitable for model-informed precision dosing in clinical routine.

## 2. Materials and Methods

### 2.1. Clinical Data and Patients

This study was a monocentric, retrospective analysis in two intensive care units of a tertiary care hospital (LMU hospital Munich) investigating VRC plasma concentrations from February 2016 to November 2019. The local institutional review board approved the study (registration number 20-168). Patients were administered a standard dose of intravenous VRC of 6 mg/kg q12h on day 1 and 4 mg/kg q12 h on the following days. The routine TDM of VRC was performed twice weekly (Monday and Thursday). VRC plasma levels meeting the following criteria were included in the analysis:

(1) Patients treated with VRC for a possible, probable or proven invasive aspergillosis according to EORTC/MSG consensus [16].
(2) Patients aged ≥18 years.
(3) Patients with at least two measured VRC concentrations.
(4) Patients with full dosing history available.

For each patient, demographic data (sex, age, weight, height, transplantation and disease), medication records (VRC amount and time of dose) and laboratory parameters, including albumin, aspartate-aminotransferase (GOT), alanine-aminotransferase (GPT), bilirubin, creatinine, glomerular filtration rate (GFR) calculated according to the Chronic Kidney Disease Epidemiology Collaboration (CKD-EPI), C-reactive protein (CRP) and Interleukin 6 (IL-6), were collected from the medical record. VRC plasma concentrations were measured with isotope-dilution liquid chromatography tandem mass spectrometry (LC-MS/MS) using a commercially available TDM kit (Chromsystems, Gräfelfing, Germany) according to a published method [17].

### 2.2. Model Selection/Literature Research

PubMed was screened for existing VRC pharmacokinetic models for adult patients until May 2021 using the following mesh terms: “voriconazole” AND “pharmacokinetics” OR “Bayesian forecasting” OR “pharmacokinetic model”. Models with CYP-Genotype as a covariate, two-stage and non-parametric approach were excluded as well as models for children and adolescence. The models were encoded, cross checked (by SK and UL) and processed in NONMEM 7.5 (Icon Development Solutions, Hanover, MD, USA).

### 2.3. Model Evaluation

The different models were compared using visual predictive checks (VPC), normalized prediction distribution errors (NPDE) and classical goodness-of-fit plots (GOF). The distribution of the NPDEs was visualized using histograms and a quantile-quantile plot. Since it can be assumed that the NPDEs follow a normal distribution, the mean and variance were calculated. The Student’s T-test and Fischer test were employed for statistical analysis.

In addition, the models were compared by calculating relative bias (rBias) and mean error (ME) as criteria for accuracy and relative root mean squared error (rRMSE) and mean absolute error (MAE) as criteria for precision according to previously used formulas [18,19]. A priori predictions were calculated from a deterministic simulation using only the patients’ covariates and the dosing histories to predict the second measured concentration. Bayesian predictions were calculated including additionally the first measured plasma concentration.

$$\mathrm{rBias}\;\left[\%\right]=\frac{1}{N}\times \overset{i}{\underset{1}{\sum }}\left(\frac{{\mathrm{predicted}\;\mathrm{concentration}}_{i}-{\mathrm{observed}\;\mathrm{concentration}}_{i}}{({\mathrm{observed}\;\mathrm{concentration}}_{i}+\mathrm{predicted}\;\mathrm{concentration})/2}\right)\times 100$$

$$\mathrm{rRMSE}\;\left[\%\right]=\sqrt{\frac{1}{N}\times \overset{i}{\underset{1}{\sum }}\left(\frac{{\left({\mathrm{predicted}\;\mathrm{concentration}}_{i}-{\mathrm{observed}\;\mathrm{concentration}}_{i}\right)}^{2}}{{\left(({\mathrm{observed}\;\mathrm{concentration}}_{i}+\mathrm{predicted}\;\mathrm{concentration})/2\right)}^{2}}\right)}\times 100\;$$

$$\mathrm{ME}\;\left[\frac{\mathrm{mg}}{L}\right]=\frac{1}{N}\times \overset{i}{\underset{1}{\sum }}\left({\mathrm{predicted}\;\mathrm{concentration}}_{i}-{\mathrm{observed}\;\mathrm{concentration}}_{i}\right)$$

$$\mathrm{MAE}\;\left[\frac{\mathrm{mg}}{L}\right]=\frac{1}{N}\times \overset{i}{\underset{1}{\sum }}\left|{\mathrm{predicted}\;\mathrm{concentration}}_{i}-{\mathrm{observed}\;\mathrm{concentration}}_{i}\right|$$

A relative bias between −20% and 20% was considered as unbiased [20]. Graphical and numerical analyses were performed using R Core Team (2022). (R: A language and environment for statistical computing. R Foundation for Statistical Computing, Vienna, Austria. URL https://www.R-project.org/, accessed on 2 February 2022.)

## 3. Results

### 3.1. Data and Patients

In total, 33 patients with two VRC plasma concentrations per patient (*n* = 66) were included in our study. The median time between the two measured samples per patient was 72 h. The median age of the patients was 44 years (range 26–67). The median SAPS II in our population at the first day of VRC therapy was 40. The median bilirubin of 0.7 mg/dL (range: 0.2 mg/dL to 7.1 mg/dL) indicates no liver dysfunction for most of the patients. A total of 20 patients were lung transplant recipients, 12 patients were admitted with the diagnosis of acute respiratory distress syndrome (ARDS) and one patient received a liver transplant. Detailed patient characteristics are displayed in Table 1.

### 3.2. Population Pharmacokinetic Models

The literature search revealed 36 potential PK models at baseline, of which only seven models were kept [4,23,24,25,26,27,28]. Models had to be excluded for the following reasons: 18 models included pharmacogenetic information, 5 models were developed for adolescents or children and 1 study used a non-parametric approach. In addition, the models by Ruiz et al. and Nomura et al. were excluded due to incomplete documentation (residual variability not reported) and three models were excluded because they considered covariates not available in our dataset (Han et al. (2010): postoperative time; Han et al. (2011): postoperative time; Lin et al.: Child–Pugh Score) [29,30,31,32,33]. Of the included models, Perez-Pitarch et al. [23] used a two-compartment model, while all other authors used one-compartment models. The covariates included were direct bilirubin (*n* = 2), body weight (*n* = 3), albumin (*n* = 2), platelets count (*n* = 2) and γ-glutamyl transferase (*n* = 1). All models included interindividual variability, but none of them included interoccasion variability.

### 3.3. Model Evaluation

**Figure 1:**
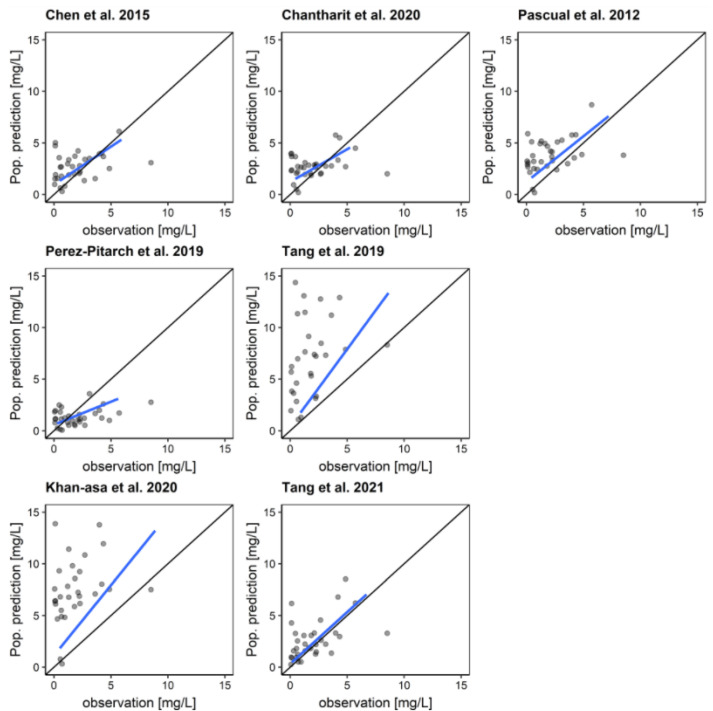
Goodness-of-fit plots for the investigated population PK models comparing population predictions with the second measured plasma concentration [4,23,24,25,26,27,28]. Black line: line of identity; blue line: linear regression line. Points: measured concentrations.

Population-based goodness-of-fit plots are shown in Figure 1. The numerical results of the NPDE assessments are shown in Table 2. NPDE and VPC plots can be found in the Supplementary Materials. The mean NPDE was significantly (*p* < 0.05) negative for five of the models, indicating a general overestimation of the measured concentrations by the respective models. The comparison of the variance by the NPDE was significantly different from 1 for the models by Perez-Pitarch et al. [23] and Tang et al., 2021 [26] (Fisher, *p* < 0.05). Apart from the model by Chantharit et al., NPDEs showed numerically a significant mischaracterization of the data by all models. This mismatch was confirmed by the VPCs (see Supplementary Materials). Goodness-of-fit plots of a priori predictions showed a wide dispersion of the measured values, with the linear regression line nearly matching the line of identity only for the model by Tang et al., 2021 [26].

### 3.4. Comparison of a Priori and Bayesian Predictions

**Figure 2:**
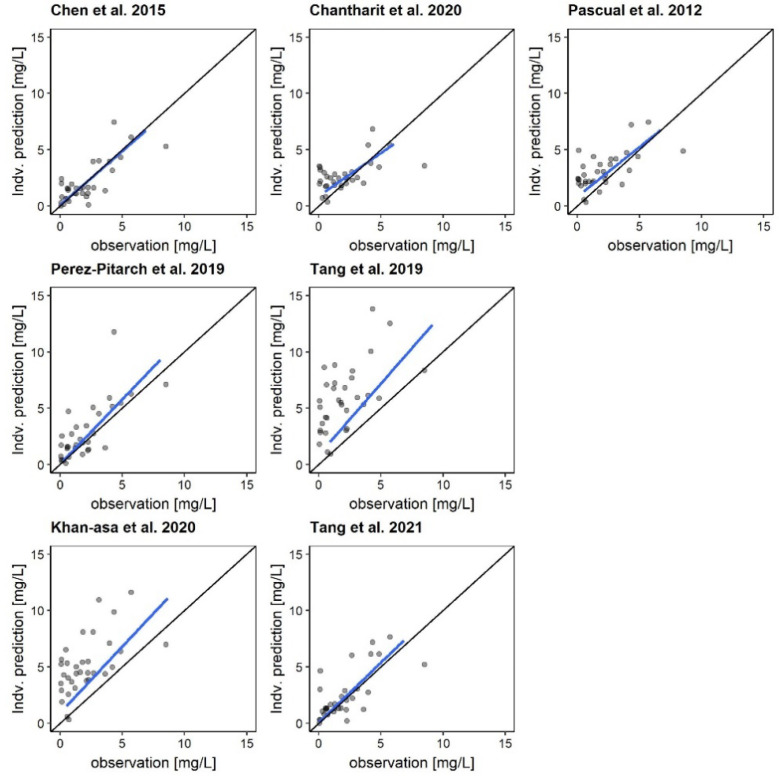
Goodness-of-fit plots for the investigated population PK models comparing individual predictions of the second measured plasma concentrations using the first plasma concentration and Bayesian forecasting [4,23,24,25,26,27,28]. Black line: line of identity; blue line: linear regression line. Points: measured concentrations.

The a priori rBias/ME and rRMSE/MAE varied markedly between the models, ranging from −15.4% to 124.6%/−0.70 mg/L to 8.01 mg/L and from 89.3% to 139.1%/1.45 to 8.11 mg/L, respectively (Table 3). The model by Perez-Pitarch et al. [23] revealed the best a priori rBias (rBias/ME: −15.4%/−0.70 mg/L). However, the precision was low for this model (rRMSE/MAE: 106.6%/1.46 mg/L). If one TDM sample was included in the prediction and the second sample was predicted using Bayesian forecasting, the median rBias remained similar (median rBias of all models a priori: 48.6%; Bayesian: 49.5%), while the median rRMSE improved slightly (median rRMSE of all models a priori: 106.6%; Bayesian: 91.4%). The model with the best predictive performance using Bayesian forecasting was the model by Chen et al. with an rBias/ME of 4.0%/−0.01 mg/L and an rRMSE/MAE of 85.0%/0.91 mg/L. The model by Chen et al. showed no structural deviation across the entire concentration range in the graphical analysis, while all other models indicated some structural error (Figure 2).

## 4. Discussion

Pharmacokinetic models are a valuable tool and increasingly important for assisting health-care professionals in dosing decisions and optimization [34,35]. The integration of patient-specific characteristics, dosing information and measured drug concentrations and the consideration of interpatient variability enable a more personalized approach to therapeutic decision making, which is especially important in vulnerable patient populations and drugs with a narrow therapeutic range such as VRC [36]. Brendel et al. revealed that only 7% of the developed pharmacokinetic models are externally validated [37]. Bayesian forecasting is becoming increasingly important in the clinical setting, providing a lot of information with little data. In general, the patient populations employed for the development of VRC models were heterogenous, ranging from children to adults with lung transplantation [30,38]. In the past, there have been several VRC PopPK models described, but only few of them have been externally evaluated. Particularly developed for intensive care patients was only the model by Ruiz et al., which had to be excluded from our analysis due of insufficient model information [32]. Farkas et al. compared three different population PK models with linear, mixed and non-linear elimination based on 519 samples from 67 patients and revealed a sufficient predictive performance for all model types [39]. Moreover, Han et al. externally validated their developed model with 52 samples from 19 patients and Chen et al. externally validated their developed model with 7 samples from 7 patients from the same study center [29,40]. To the best of our knowledge, the present study is the first external and systematic evaluation of the predictive performance of several VRC pharmacokinetic models in an independent critically ill population.

In our study, a priori predictions were imprecise and subject to considerable bias for most models, indicating that patients’ covariates alone were not sufficient to predict future VRC plasma concentrations accurately. The calculation of the NPDEs underlined the inaccuracy of the models for our patient population, showing for most of them a general overestimation. However, the predictions were slightly more precise using one measured plasma concentration. The best predictive performance including one sample in the predictions revealed the model by Chen et al., which is a one-compartment model including bilirubin in serum as a covariate on VRC clearance [24]. Comparing the results of our evaluation study with previous evaluation studies, a relatively high degree of imprecision and a considerable bias for most models is noticeable. For example, Broeker et al. evaluated population pharmacokinetic models of vancomycin using Bayesian forecasting with the integration of one measured plasma concentration which resulted in a median rRMSE of 32.5% across all investigated models compared to the lowest rRMSE of 83.5% in our study [19]. Several reasons could explain this result and might have impacted the results of our study: first, a real-world dataset using intermittent infusion was employed in our study, which entails an increased rate of documentation inaccuracies compared to prospective study conditions. If a “trough” concentration has been sampled, e.g., after the start of an infusion, a significant error is to be expected [41]. However, no clear outliers were identified in the graphical analysis. Furthermore, it should be noted that the time interval from the first to the second measured concentration was quite long, with a median time of 72 h. Pathophysiological processes altering the pharmacokinetics in the meantime might not be adequately reflected. In the case of very long treatment durations, a “prediction update” might therefore be carried out by re-sampling. In general, it would be expectable that the predictions become more precise when more than one TDM sample is integrated into the predictions [39]. The investigation of alternative Bayesian scenarios was unfortunately not possible due to insufficient data. Second, the predictive performance of the models should be analyzed against the residual errors of the models examined. Here, an extremely wide range was observed between the models with very high residual errors in some cases (Khan-asa et al. additive error: 2.67 mg/L, Chantharit et al. proportional error: 58% coefficient of variation), indicating a high proportion of unexplained variability [26,27,28]. In our investigation, such high residual errors in the models seem to prevent Bayesian forecasting from predicting the second measured concentrations using the first reliably. Third, none of the investigated models included non-linear elimination, which is surprising since elimination was described as non-linear several times [32,42]. However, since non-linear VRC elimination occurs mainly at very high doses (>10 mg/kg/d), it can be assumed that this may not have had a major impact on the predictive performance in our population [39]. Since none of the models showed a very good model performance, it should be investigated whether models that incorporate pharmacogenetic information could achieve a significantly higher precision in the future, which would imply assessing this information in routine clinical practice for the use of model-informed precision dosing.

It remains to be discussed whether the evaluated models are applicable for routine use and model-informed precision dosing. Farkas et al. analyzed the predictive performance of linear, mixed and non-linear elimination models using Bayesian methods and found mean squared error values of 2.1, 4.98 and 4.97 mg^2^/L^2^ [39]. The authors concluded that the models are applicable in clinical routine. Even if the quantities are not directly comparable, the MAE of <1 mg/L of the model by Chen et al. might allow a reasonable applicability, since the target range for VRC trough concentrations of 1–5.5 mg/L allows some uncertainty. However, in our opinion, the high imprecision should be considered and at most, TDM measurements could be supported, but not replaced.

Our study has some limitations: The results cannot necessarily be transferred from critically ill patients to other populations. Further evaluations are needed in other patient populations. It was beyond the scope of this analysis to adapt, manipulate or optimize existing models. Future studies might investigate the performance of VRC models including pharmacogenetic covariates or using the $PRIOR subroutine in NONMEM to re-estimate existing models. Another valuable option might be physiologically based pharmacokinetic (PBPK) models to better represent the complexity of VRC pharmacokinetics in intensive care patients.

To summarize, our systematic evaluation compared PK models without pharmacogenetic covariates for its suitability for Bayesian forecasting and identified the Bayesian predictions of the model by Chen et al. as unbiased and with the highest degree of precision. The precision of all models, however, was rather low. Future studies should investigate whether PopPK models that incorporate pharmacogenetic information yield better predictive performance.

## Figures and Tables

**Table 1:** Patient characteristics at initiation of VRC therapy.

	*n*
Number of patients	33
- Male	23
- Female	10
Diseases	
- Lung transplantation	20
- ARDS	12
- Liver transplantation	1
	**Median (Range)**
Age (years)	44 (26–67)
Weight (kg)	71 (40–120)
Height (cm)	175 (160–188)
BMI (kg/m^2^)	22.4 (13.1–37.0)
Voriconazole plasma concentration sample 1 (mg/L)	1.51 (0.08–8.89)
Voriconazole plasma concentration sample 2 (mg/L)	1.62 (0.06–8.51)
SAPS II	40 (23–77)
LBW (kg)	45.9 (37.9–63.6)
IL6 (pg/mL)	39 (3.4–415)
CRP (mg/dL)	8.0 (0.3–46.6)
Creatinine (mg/dL)	0.9 (0.3–3.1)
GFR-CKD-EPI (mL/min)	109 (20–206)
Albumin (mg/dL)	2.3 (1.8–3.0)
GOT (U/L)	30 (8–7000)
GPT (U/L)	29 (5–918)
Bilirubin (mg/dL)	0.7 (0.2–7.1)

Abbreviations: BMI: body mass index; CRP: C-reactive protein; GFR-CKD-EPI: glomerular filtration rate according to [21]; GOT: glutamate-oxalacetate-transaminase; GPT: glutamate-pyruvate-transaminase; IL-6: interleukin 6; LBW: lean body weight; SAPS: Simplified Acute Physiology Score [22].

**Table 2:** Mean and variance of the NDPE distribution.

	Mean	*p* (*t*-Test)	Variance	*p* (Fisher Variance)
Chen et al., 2015 [24]	−0.3247	0.03	1.0122	1
Chantharit et al., 2020 [28]	−0.0777	1	1.3098	0.286
Khan-asa et al., 2020 [27]	−1.0333	<0.001	0.7391	0.3416
Pascual et al., 2012 [4]	−0.7567	<0.001	1.0699	1
Perez-Pitarch et al., 2019 [23]	0.1031	1	3.2898	<0.001
Tang et al., 2019 [25]	−1.2824	<0.001	0.9115	1
Tang et al., 2021 [26]	−0.5025	0.04	2.5568	<0.001

**Table 3:** Performance metrics of seven evaluated voriconazole models.

	rRMSE (%)	rBias (%)	ME (mg/L)	MAE (mg/L)
	*A priori/Bayes*	*A priori/Bayes*	*A priori/Bayes*	*A priori/Bayes*
Chen et al., 2015 [24]	96.9/85.0	48.2/4.0	0.67/−0.01	1.45/0.91
Chantharit et al., 2020 [28]	97.7/91.4	48.6/49.5	0.70/0.66	1.54/1.29
Khan-asa et al., 2020 [28]	139.1/115.9	124.6/95.6	8.01/3.10	8.11/3.21
Pascual et al., 2012 [4]	108.5/96.7	74.3/59.8	1.78/1.06	2.26/1.54
Perez-Pitarch et al., 2019 [23]	106.6/83.7	−15.4/40.6	−0.70/0.81	1.46/1.24
Tang et al., 2019 [25]	135.7/122.1	124.4/106.9	7.15/3.79	7.16/3.80
Tang et al., 2021 [26]	89.3/83.5	42.9/20.2	0.66/0.41	1.45/1.13

Abbreviations: rRMSE: relative root mean squared error; rBias: relative Bias; ME: mean error; MAE: mean absolute error.

## Data Availability

Not applicable.

## Supplementary Materials

The following supporting information can be downloaded at: https://www.mdpi.com/article/10.3390/pharmaceutics14091920/s1, Figure S1: Graphical evaluation of the model by Chen et al.; Figure S2: Graphical evaluation of the model by Chantharit et al.; Figure S3: Graphical evaluation of the model by Khan-asa et al.; Figure S4: Graphical evaluation of the model by Pascual et al.; Figure S5: Graphical evaluation of the model by Perez-Pitarch et al.; Figure S6: Graphical evaluation of the model by Tang et al., 2019; Figure S7: Graphical evaluation of the model by Tang et al., 2021.
