# Supplementary material for: Systematic Evaluation of Voriconazole Pharmacokinetic Models without Pharmacogenetic Information for Bayesian Forecasting in Critically Ill Patients
Source: Pharmaceutics. 2022 Sep 10;14(9):1920. doi: 10.3390/pharmaceutics14091920 (PMC9505877; doi:10.3390/pharmaceutics14091920)
Supplement: Supplementary file 1 [file pharmaceutics-14-01920-s001.zip › pharmaceutics-1865124-supplementary.pdf]

Article

Systematic Evaluation of Voriconazole Pharmacokinetic Models without Pharmacogenetic Information for Bayesian Forecasting in Critically Ill Patients

Simon Kallee[1], Christina Scharf[1], Lea Marie Schatz[2], Michael Paal[3], Michael Vogeser[3], Michael Irlbeck[1], Johannes Zander[4], Michael Zoller[1], Uwe Liebchen[1]*

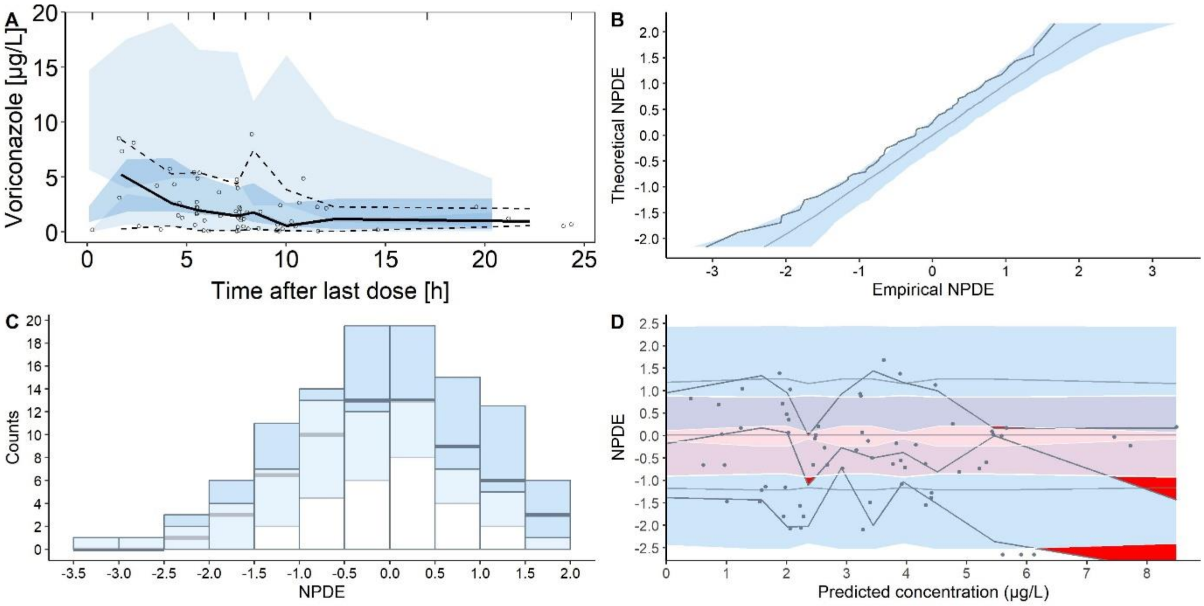

Figure S1. Graphical evaluation of the model by Chen et al. **A:** Visual predictive check. Light blue shaded regions: 90% prediction bands associated with the 5th and 95th percentiles of pharmacokinetic (PK) model simulations (N=1000 iterations). Dark blue region: 90% prediction band associated with the 50th percentile of PK model simulations. Dashed lines: 5th and 95th percentiles of observed data; solid line: 50th percentile of observed data. Dots: observed data. **B**: Quantile-Quantile plot. Theoretical Normalised Prediction Distribution Error (NPDE) vs empirical NPDE; light blue shaded region: Simulation based 95% prediction interval (N=1000 iterations) with 50th percentile (straight solid line); solid line: 50th percentile of the empirical NPDE; **C:** Theoretical vs empirical NPDE; light blue shaded region: 90% simulation based (N=1000) prediction interval; solid line: 50th percentile; white histogram: Empirical NPDE; **D:** Theoretical and empirical NPDE distribution vs. predicted voriconazole concentration. dots: observed concentrations; lines: 5th, 50th and 95th percentiles of observed concentrations; blue areas: 90% prediction intervals of the 5th and 95th percentiles; light red area: 90% prediction interval of the 50th percentile; the dark red area indicates a mismatch of the model.

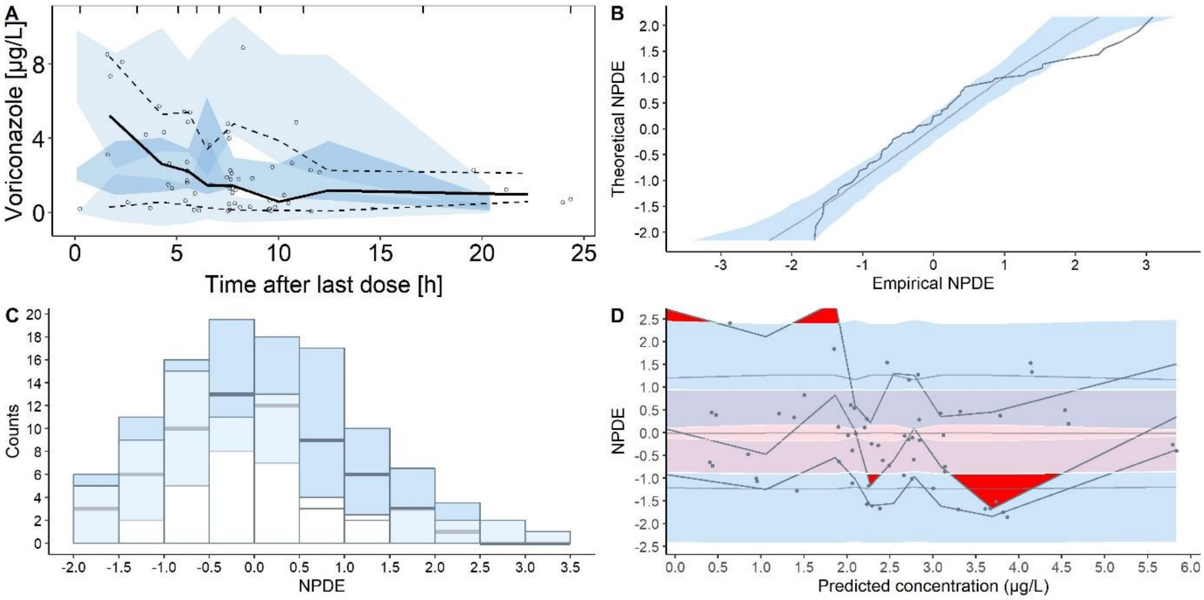

Figure S2. Graphical evaluation of the model by Chantharit et al. **A:** Visual predictive check. Light blue shaded regions: 90% prediction bands associated with the 5th and 95th percentiles of pharmacokinetic (PK) model simulations (N=1000 iterations). Dark blue region: 90% prediction band associated with the 50th percentile of PK model simulations. Dashed lines: 5th and 95th percentiles of observed data; solid line: 50th percentile of observed data. Dots: observed data. **B**: Quantile-Quantile plot. Theoretical Normalised Prediction Distribution Error (NPDE) vs empirical NPDE; light blue shaded region: Simulation based 95% prediction interval (N=1000 iterations) with 50th percentile (straight solid line); solid line: 50th percentile of the empirical NPDE; **C:** Theoretical vs empirical NPDE; light blue shaded region: 90% simulation based (N=1000) prediction interval; solid line: 50th percentile; white histogram: Empirical NPDE; **D:** Theoretical and empirical NPDE distribution vs. predicted voriconazole concentration. dots: observed concentrations; lines: 5th, 50th and 95th percentiles of observed concentrations; blue areas: 90% prediction intervals of the 5th and 95th percentiles; light red area: 90% prediction interval of the 50th percentile; the dark red area indicates a mismatch of the model.

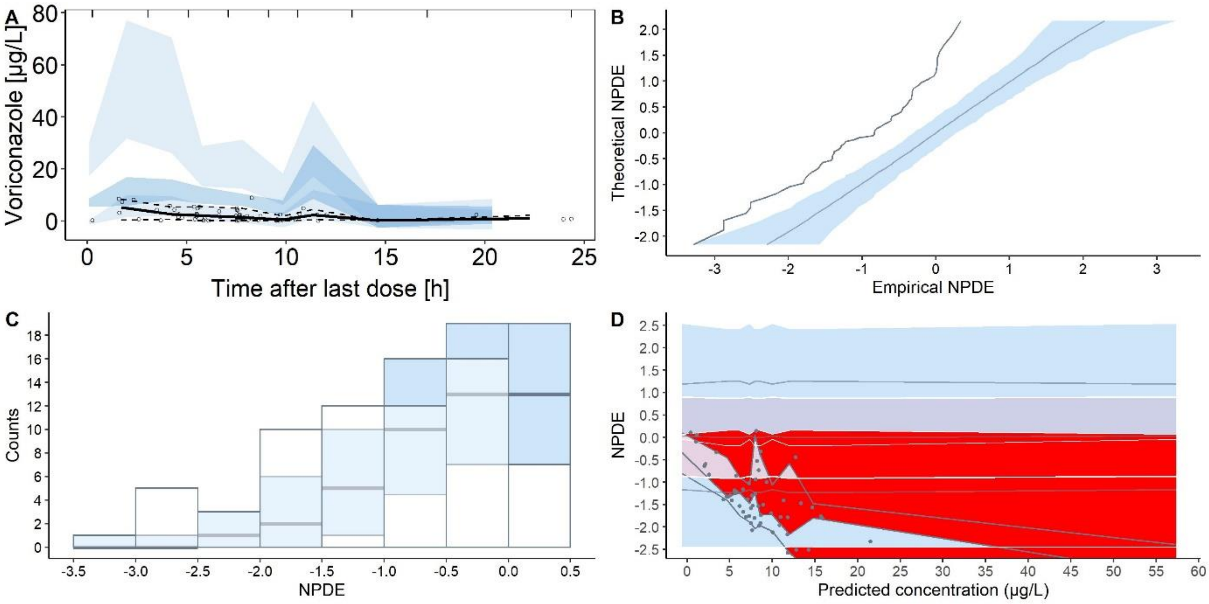

Figure S3. Graphical evaluation of the model by Khan-asa et al.. **A:** Visual predictive check. Light blue shaded regions: 90% prediction bands associated with the 5th and 95th percentiles of pharmacokinetic (PK) model simulations (N=1000 iterations). Dark blue region: 90% prediction band associated with the 50th percentile of PK model simulations. Dashed lines: 5th and 95th percentiles of observed data; solid line: 50th percentile of observed data. Dots: observed data. **B**: Quantile-Quantile plot. Theoretical Normalised Prediction Distribution Error (NPDE) vs empirical NPDE; light blue shaded region: Simulation based 95% prediction interval (N=1000 iterations) with 50th percentile (straight solid line); solid line: 50th percentile of the empirical NPDE; **C:** Theoretical vs empirical NPDE; light blue shaded region: 90% simulation based (N=1000) prediction interval; solid line: 50th percentile; white histogram: Empirical NPDE; **D:** Theoretical and empirical

NPDE distribution vs. predicted voriconazole concentration. dots: observed concentrations; lines: 5th, 50th and 95th percentiles of observed concentrations; blue areas: 90% prediction intervals of the 5th and 95th percentiles; light red area: 90% prediction interval of the 50th percentile; the dark red area indicates a mismatch of the model.

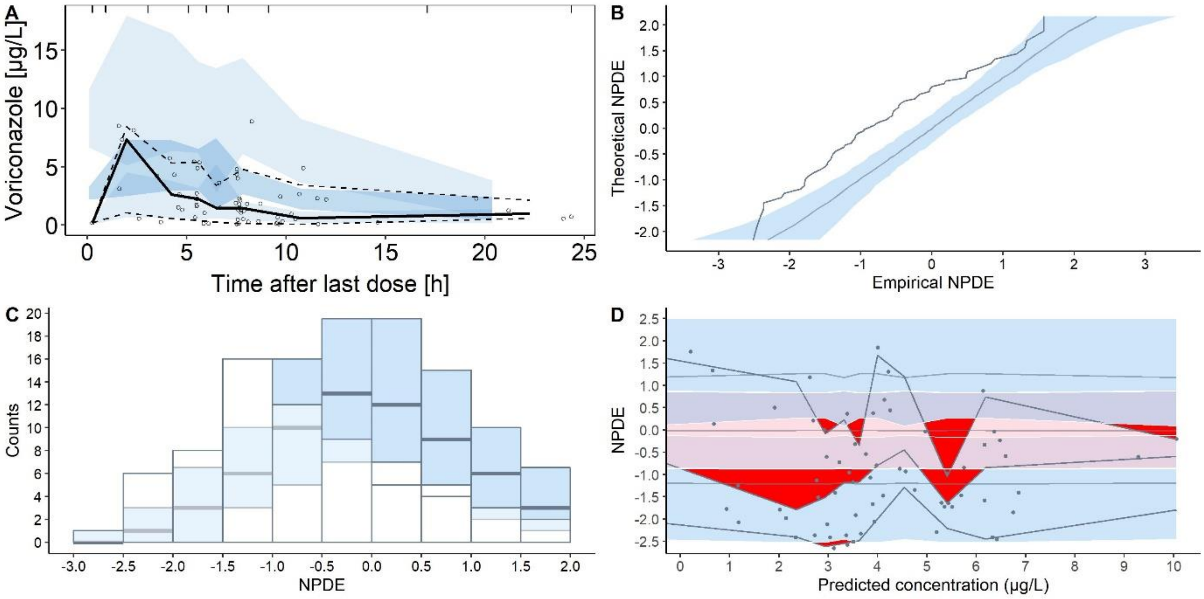

Figure S4. Graphical evaluation of the model by Pascual et al. **A:** Visual predictive check. Light blue shaded regions: 90% prediction bands associated with the 5th and 95th percentiles of pharmacokinetic (PK) model simulations (N=1000 iterations). Dark blue region: 90% prediction band associated with the 50th percentile of PK model simulations. Dashed lines: 5th and 95th percentiles of observed data; solid line: 50th percentile of observed data. Dots: observed data. **B**: Quantile-Quantile plot. Theoretical Normalised Prediction Distribution Error (NPDE) vs empirical NPDE; light blue shaded region: Simulation based 95% prediction interval (N=1000 iterations) with 50th percentile (straight solid line); solid line: 50th percentile of the empirical NPDE; **C:** Theoretical vs empirical NPDE; light blue shaded region: 90% simulation based (N=1000) prediction interval; solid line: 50th percentile; white histogram: Empirical NPDE; **D:** Theoretical and empirical NPDE distribution vs. predicted voriconazole concentration. dots: observed concentrations; lines: 5th, 50th and 95th percentiles of observed concentrations; blue areas: 90% prediction intervals of the 5th and 95th percentiles; light red area: 90% prediction interval of the 50th percentile; the dark red area indicates a mismatch of the model.

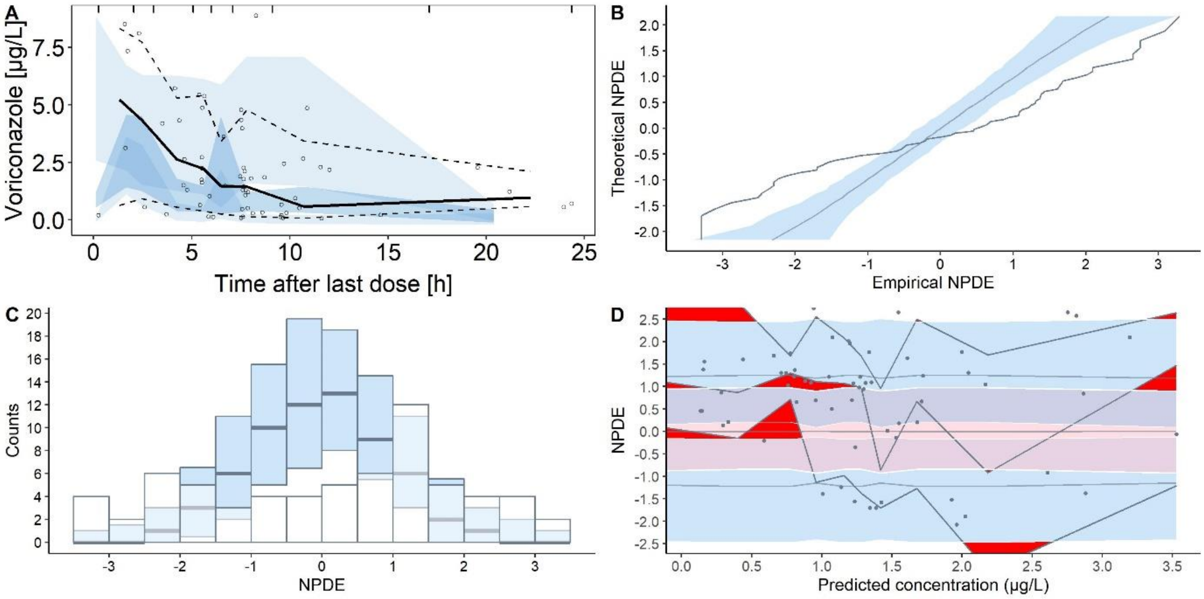

Figure S5. Graphical evaluation of the model by Perez-Pitarch et al.. **A:** Visual predictive check. Light blue shaded regions: 90% prediction bands associated with the 5th and 95th percentiles of pharmacokinetic (PK) model simulations (N=1000

iterations). Dark blue region: 90% prediction band associated with the 50th percentile of PK model simulations. Dashed lines: 5th and 95th percentiles of observed data; solid line: 50th percentile of observed data. Dots: observed data. **B**: Quantile-Quantile plot. Theoretical Normalised Prediction Distribution Error (NPDE) vs empirical NPDE; light blue shaded region: Simulation based 95% prediction interval (N=1000 iterations) with 50th percentile (straight solid line); solid line: 50th percentile of the empirical NPDE; **C:** Theoretical vs empirical NPDE; light blue shaded region: 90% simulation based (N=1000) prediction interval; solid line: 50th percentile; white histogram: Empirical NPDE; **D:** Theoretical and empirical NPDE distribution vs. predicted voriconazole concentration. dots: observed concentrations; lines: 5th, 50th and 95th percentiles of observed concentrations; blue areas: 90% prediction intervals of the 5th and 95th percentiles; light red area: 90% prediction interval of the 50th percentile; the dark red area indicates a mismatch of the model.

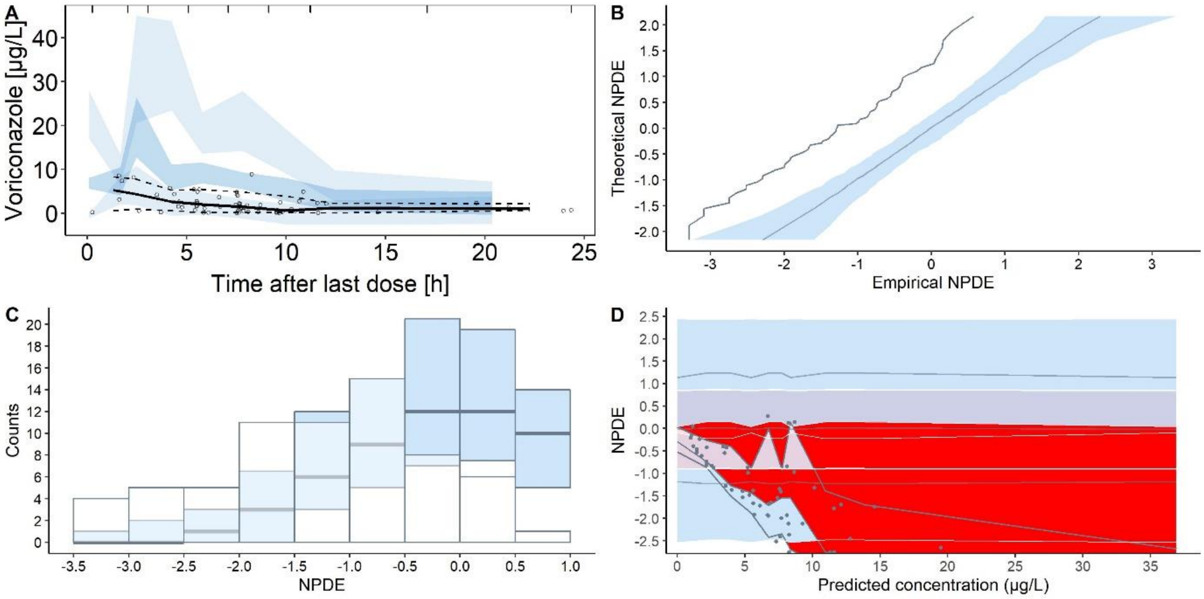

Figure S6. Graphical evaluation of the model by Tang et al. 2019. **A:** Visual predictive check. Light blue shaded regions: 90% prediction bands associated with the 5th and 95th percentiles of pharmacokinetic (PK) model simulations (N=1000 iterations). Dark blue region: 90% prediction band associated with the 50th percentile of PK model simulations. Dashed lines: 5th and 95th percentiles of observed data; solid line: 50th percentile of observed data. Dots: observed data. **B**: Quantile-Quantile plot. Theoretical Normalised Prediction Distribution Error (NPDE) vs empirical NPDE; light blue shaded region: Simulation based 95% prediction interval (N=1000 iterations) with 50th percentile (straight solid line); solid line: 50th percentile of the empirical NPDE; **C:** Theoretical vs empirical NPDE; light blue shaded region: 90% simulation based (N=1000) prediction interval; solid line: 50th percentile; white histogram: Empirical NPDE; **D:** Theoretical and empirical NPDE distribution vs. predicted voriconazole concentration. dots: observed concentrations; lines: 5th, 50th and 95th percentiles of observed concentrations; blue areas: 90% prediction intervals of the 5th and 95th percentiles; light red area: 90% prediction interval of the 50th percentile; the dark red area indicates a mismatch of the model.

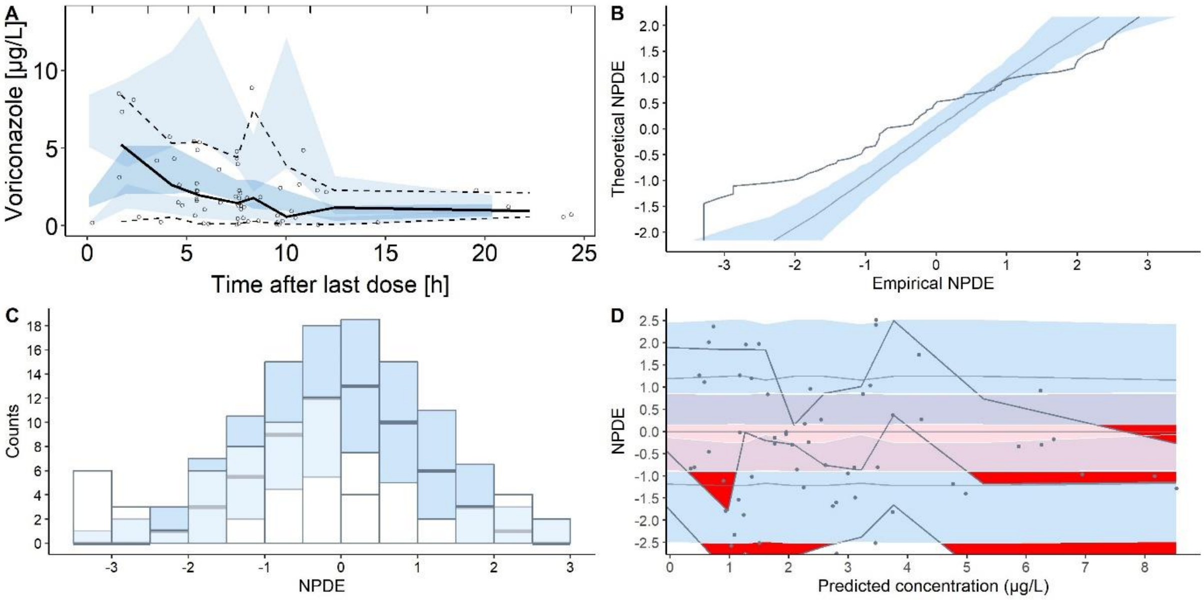

Figure S7. Graphical evaluation of the model by Tang et al. 2021. **A:** Visual predictive check. Light blue shaded regions: 90% prediction bands associated with the 5th and 95th percentiles of pharmacokinetic (PK) model simulations (N=1000 iterations). Dark blue region: 90% prediction band associated with the 50th percentile of PK model simulations. Dashed lines: 5th and 95th percentiles of observed data; solid line: 50th percentile of observed data. Dots: observed data. **B**: Quantile-Quantile plot. Theoretical Normalised Prediction Distribution Error (NPDE) vs empirical NPDE; light blue shaded region: Simulation based 95% prediction interval (N=1000 iterations) with 50th percentile (straight solid line); solid line: 50th percentile of the empirical NPDE; **C:** Theoretical vs empirical NPDE; light blue shaded region: 90% simulation based (N=1000) prediction interval; solid line: 50th percentile; white histogram: Empirical NPDE; **D:** Theoretical and empirical NPDE distribution vs. predicted voriconazole concentration. dots: observed concentrations; lines: 5th, 50th and 95th percentiles of observed concentrations; blue areas: 90% prediction intervals of the 5th and 95th percentiles; light red area: 90% prediction interval of the 50th percentile; the dark red area indicates a mismatch of the model.
